# Ethnobotanical survey of medicinal plants used in the treatment of animal diarrhoea in Plateau State, Nigeria

**DOI:** 10.1186/1746-6148-7-36

**Published:** 2011-07-11

**Authors:** Nkechi V Offiah, Sunday Makama, Ishaku L Elisha, Micah S Makoshi, Jurbe G Gotep, Christiana J Dawurung, Olusola O Oladipo, Ann S Lohlum, David Shamaki

**Affiliations:** 1 National Veterinary Research Institute, P.M.B. 01, Vom, Plateau State, Nigeria; 2 School of Veterinary Medicine, Faculty of Medical Sciences, The University of the West Indies, St. Augustine, Trinidad & Tobago

## Abstract

**Background:**

The use of medicinal plants in the treatment of diseases has generated renewed interest in recent times, as herbal preparations are increasingly being used in both human and animal healthcare systems. Diarrhoea is one of the common clinical signs of gastrointestinal disorders caused by both infectious and non-infectious agents and an important livestock debilitating condition. Plateau State is rich in savannah and forest vegetations and home to a vast collection of plants upheld in folklore as having useful medicinal applications. There is however scarcity of documented information on the medicinal plants used in the treatment of animal diarrhoea in the state, thus the need for this survey. Ten (10) out of 17 Local Government Areas (LGAs), spread across the three senatorial zones were selected. Farmers were interviewed using well structured, open-ended questionnaire and guided dialogue techniques between October and December 2010. Medicinal plants reported to be effective in diarrhoea management were collected using the guided field-walk method for identification and authentication.

**Results:**

A total of 248 questionnaires were completed, out of which 207 respondents (83.47%) acknowledged the use of herbs in diarrhoea management, while 41 (16.53%) do not use herbs or apply other traditional methods in the treatment of diarrhoea in their animals. Medicinal plants cited as beneficial in the treatment of animal diarrhoea numbered 132, from which 57(43.18%) were scientifically identified and classified into 25 plant families with the families Fabaceae (21%) and Combretaceae (14.04%) having the highest occurrence. The plant parts mostly used in antidiarrhoeal herbal preparations are the leaves (43.86%) followed by the stem bark (29.82%). The herbal preparations are usually administered orally.

**Conclusion:**

Rural communities in Plateau State are a rich source of information on medicinal plants as revealed in this survey. There is need to scientifically ascertain the authenticity of the claimed antidiarrhoeal properties of these plants and perhaps develop more readily available alternatives in the treatment of diarrhoea.

## Background

Herbal medicine has long been recognized as one of the oldest forms of remedies used by humans [1]. Many people in developing countries still rely on traditional healing practices and medicinal plants for their daily healthcare needs, in spite of the advancement in modern medicine [2]. There is abundant undocumented traditional knowledge of herbal remedies used to treat diseases in most cultures [3]. Different traditional healing practices worldwide are designed for either therapeutic or prophylactic use in human or animal diseases [4,5]. Several studies carried out in Africa, Asia, Europe, Latin America and North America show that plants are routinely used as remedy for animal diseases [6-11]. Historically, it is documented that humans utilize the same herbal preparations that they use to treat their sick animals [1]. In Nigeria, farmers are known to treat animal diseases with herbs and other traditional medical practices before the advent of orthodox medicine [12]. Traditional medical and veterinary practices remain relevant and vital in many areas in Nigeria due to absence or inadequate provision of modern medical services particularly in rural areas [13].

Ethnoveterinary medical practice is widespread among herdsmen and native livestock producers in northern Nigeria. Traditional remedies in this area include plant extracts from different plant parts [14]. Herdsmen in non industrialized nations of the world still use medicinal plants for the treatment of livestock diseases including diarrhoea, either due to lack of access to trained veterinarians and high cost of orthodox medicines, or the held belief that herbal remedies are more efficacious [15].

While diarrhoea is not intrinsically considered a disease, but rather a sign of other health problems in livestock caused by infectious and non - infectious agents, it is still the most common and costly condition affecting livestock [16]. The use of herbal drugs in the treatment of gastrointestinal disorders including diarrhoea is a common practice in many African countries [17] and is usually preferred because it is a cheaper alternative. The herbal drugs contain multiple constituents such as alkaloids, glycoside, flavonoids, terpenes, tannins etc. These constituents have effect-enhancing and/or side effect-neutralizing potential [18-20], and herbal remedies are considered relatively safe in prolonged use [19].

Plateau State, is Nigeria's foremost tourists' destination located strategically in the middle-belt zone, between latitude 80°24' North and longitude 80°32' and 100°38' East, and covers an area of 26,899 Sq. Km, with a population of 3, 206, 531 people [21]. Plateau State has a livestock population of 964,188 sheep, 1,865,805 goats and 1.07 million cattle [22]. A great proportion of the rural population in the state are non literates who keep livestock as a source of income [23].

Indigenous knowledge system and practices is defined as a body of knowledge that develops within a given community through observation and real life experiences over a period of time, communicated orally or otherwise from one generation to the other with the ultimate aim of moulding its thought for the sole purpose of ensuring survival and progress [24]. One of the vital applications of indigenous knowledge systems and practices is in the human and animal health care [24]. There is however a dearth of information with respect to traditional knowledge system and practices in Plateau state, due to rural-urban migration, substitution of traditional with modern practices and preference for orthodox drugs, or outright disregard for traditional practices. The population of the older generation is fast depleting due to death in Nigeria where there is a declining life expectancy projected at 48 years [25]. There is therefore an urgent need for survey and documentation of medicinal plants useful in the management of diarrhoea and in fact other diseases. In view of the many challenges and prospects mentioned above, this investigation was undertaken to provide information on the medicinal plants used by farmers in managing animal diarrhoea in Plateau State of Nigeria.

## Methods

**Figure 1:**
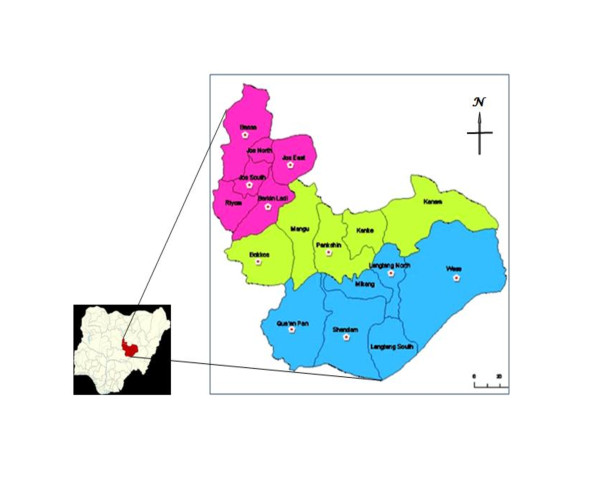
**Map of Plateau state, Nigeria showing LGAs visited (****): Pink-north, Green-central and Blue-southern geopolitical zones**. Bassa (1), Jos South (2), Jos East (3), Barkin-Ladi (4), Bokkos (5), Pankshin (6), Qu’an Pan (7), Shendam (8), Langtang North (9) and Wase (10) LGAs.

The survey was carried out in 10 selected Local Government Areas (LGAs) of Plateau state (Figure 1), which spread across the three senatorial zones based on cultural similarity and diversity during the months of October, November and December 2010. Letters seeking for assistance and cooperation of the Local Government Agricultural Departments in mobilizing livestock farmers and community leaders were written. The LGAs surveyed are Bassa, Jos East, Jos South and Barkin Ladi in the northern zone; Bokkos and Pankshin in the central zone, and; Langtang North, Shendam, Qua'an-Pan and Wase in the southern zone. These selected LGAs have a high cattle population and are favourable for livestock production and have a high population of pastoralists or natives who keep livestock [23].

Most of the rural communities surveyed have limited access to modern veterinary services largely due to bad roads. Livestock healthcare services are usually rendered by quacks that lack proper veterinary training. Plateau state has over 40 ethno-linguistic groups, with some of the tribes being Afizere, Berom, Goemai, Irigwe, Kwalla, Mwaghavul, Ngas, Ron-Kulere and Taroh [26]. These ethnic groups are predominantly farmers and have similar cultural and traditional ways of life [26]. Hausa language is a common medium of communication among these tribes like in most parts of northern Nigeria.

A well structured, open-ended questionnaire and guided dialogue techniques [24] were used to interview pastoralists and native livestock farmers. The questionnaire was designed by the team based on the needed information and validated by the epidemiology and extension units of the institute. The survey team was made up of 5-veterinarians, 1-pharmacist, 1- pharmacologist, and 2-trained veterinary extension officers. The team was randomly divided into two on a rotational basis, with one extension officer in each group at any given time. The interview was conducted in English, Hausa, Fulfulde and on rare occasions in the local dialect. Certain incentives were given to stimulate cooperation and active participation in the survey. These included free consultancy services by the veterinarians, remuneration in some instances for field staff and the promise to organize seminar for the communities visited after the conclusion of the research.

The respondents who consented to participate in the survey were asked to share their knowledge and experiences on the medicinal plants used in their communities to manage diarrhoea. Information was received on part(s) of the plant used, methods of herbal preparation, mode of administration, dosage estimation, the effectiveness of the herbal remedy and adverse effects observed. The conversation was built on trust, with the clear understanding of the aim of the survey [27]. Plants claimed to be beneficial in the treatment of diarrhoea were collected based on the guided field-walk method [15]. The plant specimens collected were pressed, labelled with their local names where available and sent to the herbarium of the Department of Biological Sciences, Ahmadu Bello University (ABU), Zaria, and identified, authenticated and voucher number assigned by Mallam U.S Gallah.

## Results

Two hundred and forty-eight questionnaires were administered during the survey. A total of 207 (83.47%) respondents admitted having used antidiarrhoeal medicinal plants or were still using them to treat their animals. Forty-one (16.55%) had no knowledge of herbs or medicinal plants used for the treatment of diarrhoea in animals. Most of the respondents especially those who still had animals were able to give adequate description of the nature of the diarrhoea often seen in their animals.

**Figure 2:**
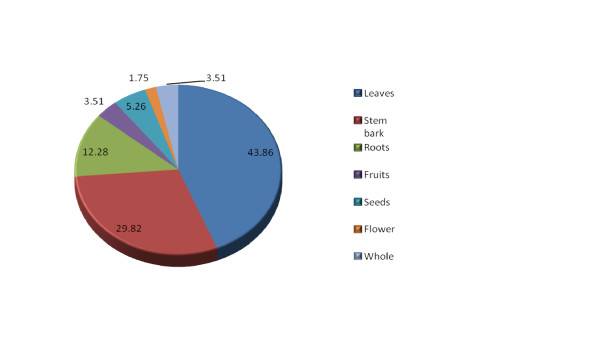
**Percentage distribution of medicinal plant parts used in the management of diarrhoea**.

**Figure 3:**
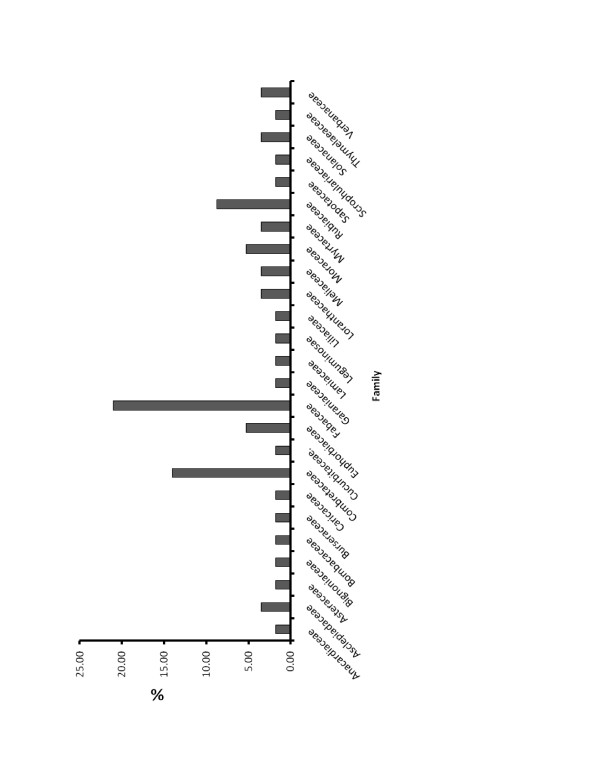
**Percentage family distribution of medicinal plants used for diarrhoea management in Plateau State**.

One hundred and thirty-two medicinal plants were collected during the survey out of which 57 were properly identified (Table 1). Their local (Nigerian) names as well as their common (English) names are presented on the same table. Various parts of these plants in use were also indicated, with the leaves being the most commonly mentioned (43.86%; Figure 2). Out of the 57 plants identified, there were 47 genera distributed among 25 families, with the families Fabaceae (21.05%). Combretaceae (14.04%) having the highest occurrences (Figure 3).

**Table 1:** Medicinal plants used by farmers for management of diarrhoea in Plateau State

						Plant parts in use						
**S/No**	***Botanical/Scientific name***	**Family**	**Common Name (English)**	Nig. Lang Name* (H; Y; I; F; Others)	**Folkloric Evidence of Use (%)**	**Leaves**	**Stem bark**	**Roots**	**Fruits**	**Seeds**	**Flower**	**Whole**

1	***Acacia albida***	Fabaceae (Mimosaceae)	Apple-ring Acacia, Winter Thorn	H: Gawo	1 (0.40%)	+	+	-	-	-	-	-
2	***Acacia sieberiana***	Fabaceae	White thorn, African laburnum	H: Farar kaya	1 (0.40%)	+	+	-	-	-	-	-
3	***Adansonia digitata,***	Bombacaceae	Baobab Tree, Judas Fruit	H: Kuka	49 (19.76%)	+	-	-	-	-	-	-
4	***Adina microcephala***	Rubiaceae	Adina, African teak	H: Madacin Rafi, Mangoron rafi	2 (0.81%)	-	+	-	-	-	-	-
5	***Aloe buettneri***	Liliaceae	West African aloe	H: Zabuwa; F: Zabuwahi	2 (0.81%)	+	-	-	-	-	-	-
6	***Anogeissus leiocarpus***	Combretaceae	African Birch	H: Marke; Y: Pako dudu, ayin	6 (2.42%)	+	+	-	-	+	-	-
7	***Azadirachta indica***	Meliaceae	Neem Tree,Nimba, Nimb	H: Dogon Yaro, Darbegiya	2 (0.81%)	+	-	-	-	-	-	-
8	***Bauhinia rufescens***	Fabaceae	Bauhinia	H: Matsagi, Kalgon Allah F: Nammare	1 (0.4%)	+	-	-	-	-	-	-
9	***Boswellia dalzielii Hutch***	Burseraceae	Frankincense tree	H: Ararabi; Hano; F: Mangalede	1 (0.40%)	-	-	-	-	-	-	-
10	***Burkea africana***	Fabaceae	Wild seringa, Rhodesian ash, seringa tree	H: Gwazan kura, Bakin makarfo F: Jill arab	1 (0.40%)	-	-	-	-	-	-	-
11	***Carica papaya***	Caricaceae	Paw-paw	H: Gwanda	3 (1.21%)	-	+	-	-	+	-	-
12	***Combretum glutinosum***	Combretaceae	-	H: kantakara, Baushe	11 (4.4%)	+	-	+	-	-	-	-
13	***Combretum lamprocarpum***	Combretaceae	-	H: Bauli; F: Buski daneehi; Zindi; Y: ajantiro	2 (0.81%)	+	-	-	-	-	-	-
14	***Combretum molle***	Combretaceae	Velvet bush/leaf willow	Gupiya (Ron)	1 (0.40%)	-	-	-	-	-	-	-
15	***Croton zambesicus***	Euphorbiaceae	Tiger bush	Berom: Lieng	1 (0.40%)	-	-	-	-	-	-	-
16	***Cucumis metuliferus***	Cucurbitaceae.	African horned cucumber or melon	Berom: Kwalchungul	14 (5.65%)	-	-	-	+	-	-	+
17	***Daniellia oliveri***	Fabaceae	African copaiba balsam tree	H: Maje, Kadaura	1 (0.41%)	+	-	-	-	-	-	-
18	***Detarium senegalensis***	Fabaceae	Sweet detar	H: Gobodo; Farar taura	3 (1.31%)	+	+	+	-	-	-	-
19	***Erythrophleum sauveolens***	Fabaceae	Ordeal tree, Sasswood tree	H: Samberu/Gwaska	1 (0.40%)	-	-	-	-	-	-	-
20	***Erythrophloem africanum***	Fabaceae	African blackwood	H:Goska; F: Naretibahi	1 (0.40%)	-	-	-	-	-	-	-
21	***Eucalyptus tereticornis***	Myrtaceae	Forest red gum	H: Turare	1 (0.40%)	-	-	-	-	-	-	-
22	***Euphorbia hirta***	Euphorbiaceae	Asthma Weed	H: Nonon kurciya; Oegendagar (Doemak)	1 (0.40%)	-	-	-	-	-	-	-
23	***Ficus ingens***	Moraceae	Red-leaved fig	F: Nunahi; Bakurahi; H: Kawuri	2 (0.81%)	+	-	-	-	-	-	-
24	***Ficus platyphylla***	Moraceae	Flake/Red Kano rubber tree	H: Gamji; F: Dundehi	2 (0.81%)	-	+	-	-	-	-	-
25	***Ficus sycomorus L***	Moraceae	Sycamore fig or fig mullberry	Baure (H)	1 (0.40%)	-	+	-	-	-	-	-
26	***Geranium spp***	Geraniaceae	Crane's bill	H: Garahunu	1 (0.40%)	-	-	-	-	-	-	-
27	***Gymnema sylvestris***	Asclepiadaceae	Miracle fruit, australian cowplant	H: Kashe zaki	1 (0.40%)	-	-	-	-	-	-	-
28	***Heeri pulcherrima***	Anacardiaceae	-	H: Hawayen zaki	1 (0.40%)	-	-	-	-	-	-	-
29	***Jatropha curcas L.***	Euphorbiaceae	Purging nut, Barbados nut	H: Bini da zugu, Halallamai Mamulu	1 (0.40%)	+	+	-	-	-	+	-
30	***Khaya senegalensis***	Meliaceae	African Mahogany	H: Madaci	43 (17.34%)	-	+	-	-	-	-	-
31	***Kigelia africana***	Bignoniaceae	Cucumber or Sausage tree	H: Nonon giwa; F: Jillarehi	2 (0.81%)	-	+	-	-	-	-	-
32	***Lasiosiphon kraussianus***	Thymelaeaceae	-	H: Shani ka sanni	1 (0.40%)	-	-	-	-	-	-	-
33	***Milletia thonningii***	Fabaceae	-	H: Tumburku; Mmaruk (Taroh)	1 (0.40%)	-	-	+	-	-	-	-
34	***Mitragyna inermis***	Rubiaceae	False abura	H: Giyayya; F: Koli	1 (0.40%)	-	-	-	-	-	-	-
35	***Moringa oleifera***	Asclepiadaceae	Drumstick Tree	H: Zogale	4 (1.61%)	+	+	-	-	+	-	-
36	***Nauclea latifolia***	Rubiaceae	African peach, Pin cushion tree	H: Tafashiya, marga; I: nwabuakonshi; Y: Egbesi plant	1 (0.40%)	-	-	-	-	-	-	-
37	***Ocimum gratissimum***	Lamiaceae	Basil, Curry leaf	H: Daidoya	1 (0.40%)	+	-	-	-	-	-	+
38	***Parkia biglobosa***	Fabaceae (Mimosaceae)	African locust bean; Monkey cutlass tree	H: Doruwa	6 (2.42%)	-	+	-	-	-	-	-
39	***Piliostigma reticulatum***	Leguminosae Caesalpiniaceae	English: camel's foot (Etkin).	H: 'kalgo; F: Batehi; Y: 'abafin' I: okpo atu	5 (2.02%)	+	-	-	-	-	-	-
40	***Piliostigma thonningii***	Fabaceae	camel's foot, monkey bread, Rhodesian bauhinia	Kalgo/Kargo (Hausa)	5 (2.02%)	+	-	-	-	-	-	-
41	***Prosopis Africana***	Fabaceae	Iron wood; Axlewood	H: Kirya; F: Kwahi	3 (1.31)	-	+	-	-	-	-	-
42	***Psidium guajava***	Myrtaceae	Guava	H: Gwaiva	16 (6.45%)	+	-	-	-	-	-	-
43	***Sarcocephalus latifolius (Smith) Bruce***	Rubiaceae	Country fig; African peach	H: Tafashi; Y: Ogbase; Hang (Berom)	3 (1.21%)	+	+	+	-	-	-	-
44	***Schwenkia americana***	Solanaceae	-	H: Dandana; F: Dupuhi; Y: Igbale odan; Collihi (Gwari)	1 (0.40%)	-	-	-	-	-	-	-
45	***Solanum dasyphyllum***	Solanaceae	-	H: Gautan Kaji; Berom: Pyalgwol	6 (2.42%)	-	-	-	+	-	-	-
46	***Spermacoce villosus***	Rubiaceae	False buttonweed	H: Goga masu Ron: Sandigis	1 (0.40%)	-	-	-	-	-	-	-
47	***Starchytarpheta angustifolia***	Verbanaceae	Devil's coach whip	H: Tsarkiyar kuusuu, Wutsiyan kadangare	2 (0.81%)	+	-	+	-	-	-	-
48	***Striga hermonthica (Del.) Benth***	Scrophulariaceae	Witchweed; purple witchweed	H: Wuta-wuta; F: Turguel	1 (0.41%)	-	-	-	-	-	-	+
49	***Tapinanthus dodoniefolius***	Loranthaceae	Mistletoe on Locust bean	H: Kauchi	1 (0.40%)	+	-	-	-	-	-	+
50	***Tapinanthus globiferus***	Loranthaceae	Mistletoe, loranthus, mulberry mistletoe	H: Kauchi	1 (0.40%)	+	-	-	-	-	-	+
51	***Terminalia avicennioides***	Combretaceae	-	H: Baushe; Y: Igiodan; I: Edo; Kpayi (Gwari)	11 (4.44%)	-	-	-	-	-	-	-
52	***Terminalia macroptera***	Combretaceae	-	H: Baushe; F: Bodi; Kung (Mwagavul)	11 (4.4%)	+	-	+	-	-	-	-
53	***Terminalia mollis***	Combretaceae	Large-leaved terminalia	Dakun (Mushere)	2 (0.81%)	-	-	+	-	-	-	-
54	***Tridax procumbens***	Combretaceae	Coat buttons	Kwalla: Magaja	1 (0.40%)	-	-	-	-	-	-	-
55	***Vernonia guineensis***	Asteraceae	-	-	1 (0.40%)	-	-	-	-	-	-	-
56	***Vitellaria paradoxa Gaertn. (Butyrospermum paradoxum)***	Sapotaceae	Sheabutter tree	H: Kadanya; I: okwuma; Y: akú malapa; Mes (Mushere)	2 (0.81%)	-	+	-	-	-	-	-
57	***Vitex doniana***	Verbanaceae	black plum	H: Dinya; F: Bodilohi (Munjiriya); I: Utakiri; Y: Ori-nla	11 (4.4%)	+	+	-	+	-	-	-

* H, Y, I, F: Hausa, Yoruba, Igbo, Fulfulde. +, Part use; -, no information on use of part.

Medicinal plants are usually prepared by soaking the fresh or dried plant parts in water and the extract administered by drenching. In few cases, the plant materials are mixed with feed and/or potash to improve palatability.

## Discussion

During this study, a total of 248 questionnaires were used to collect information from both semi-literate and non literate livestock farmers. The respondents comprised of individual livestock farmers having mainly backyard farms made up of heterogeneous species of animals and practicing the semi-intensive management system. Poultry farmers who keep local chickens manage them on free range system while the exotic breeds are reared under intensive system. From table 1, *Adansonia digitata *and *Khaya senegalensis *were the most common plants reported to be used in the management of diarrhoea in livestock with frequencies of 49 (19.76%) and 43 (17.34%) respectively. *Adansonia digitata *is very common in the northern parts of Nigeria, and earlier works had published its application in diarrhoea, malaria and cough [28,29]. The anthelminthic effect of *Khaya senegalensis *(mahogany) has been reported [30], which may justify its use in diarrhoea management. Only *Cucumis metuliferus *and *Solanum dasyphyllum *were mentioned as herbal remedies used in diarrhoea management in poultry. There are claims that the fruits of the non-bitter *Cucumis metuliferus *are effective in the management of HIV/AIDS positive patients in Plateau State, Nigeria [31], and the seeds are reported to have worm expellant properties [32].

The family Fabaceae is the most common family reported in this study, having 12 members (Figure 3), similar to the observation made by Appidi et al [33]. This observation is however, different from that of Yinegar et al. [8] who in an ethnoveterinary plant survey in Ethiopia reported Asteraceae family as the highest, followed by Solanaceae, with Fabaceae and Lamiaceae being third. This difference may be due to the fact that their survey was not narrowed to diarrhoea but also on medicinal plants used in all animal diseases. Appidi et al. [33] corroborated our findings suggesting that the Fabaceae family are more likely to have antidiarrhoeal effect than plant from other families. The Fabaceae family contains many genera that have been shown to be useful in the treatment of many other ailments besides diarrhoea [8,34]. It was also observed that the leaves (43.86%) constitute the most plant parts used, followed by the stem barks (29.82%) as shown in figure 2. Leaves are sometimes used in combination with other plant parts as reported by Ayyanar and Ignacimuthu [35]. Other indigenous populations have indicated preference for the use of leaves in the preparations of herbal medicines [35] because it is more convenient collecting leaves than root parts, flowers and fruits etc. [36]. However, some authors have reported that roots are more commonly collected plant parts in Ethnoveterinary practice [8,37,38]. Scientifically, leaves are actively involved in photosynthesis and the production of metabolites [39], thus, the numerous constituents found in leaves could explain their efficacy in the treatment of various ailments in both humans and animals. From the conservation point of view, collection of leaves for herbal preparations could be regarded as sustainable so long as some leaves are left on the parent plant [8]. This is opposed to the collection of roots which could be a severe threat for rare and slowly producing plants.

The herbal remedies were often prepared by pounding either the fresh or dried parts of the plants followed by either soaking or boiling them in water, and the infusions or decoctions administered by drenching agreeing with the observation of Ermias et al. [40]. Sometimes, the plant portions are mixed with the animal's feed and fed to the animals or mixed with potash (kanwa) or salt and given to the animals to lick. Poonam and Singh [41] reported the use of enhancers such as honey, cow/goat's milk, sugar, ghee, salt, boiled rice and butter milk to improve the palatability and medicinal property of certain remedies by the Kani traditional healers of India. The dosages often administered varied with the parts of the plants used and the mode of preparation. However most farmers administer the preparations once or twice a day for 3 to 5 days, or keep treating until the animal recovers. Full recovery is confirmed when the animals resume feeding and activities. Most respondents claimed that their herbal remedies were efficacious and produced complete healing without adverse effects.

There are documented scientific publications validating the antidiarrhoeal effect of some of the plants listed in Table 1 either using castor oil induced diarrhoea study in rats or mice and/or antimicrobial activity of extracts [17,42-44].

The problem of inconsistent dosage regimen and unwillingness to part with indigenous knowledge was experienced by the authors in the field. The latter seems to be a common experience of researchers conducting ethnobotanical surveys [45,46] and therefore a significant basis for conducting such surveys. This is because the custodians of indigenous knowledge of herbal remedies do not usually document their practices; hence transfer of knowledge to their protégés becomes difficult following their demise.

To the best our knowledge, this is the first report of herbal remedies used in the management of diarrhoea in livestock and poultry in Plateau State. This study will form the basis for evaluating the phytochemical and biological activities of the selected herbal remedies used in animal diarrhoea. Overall, the plants identified as herbal remedies in the management of diarrhoea present considerable potential for further scientific research which may lead to the discovery of newer and perhaps safer drugs.

## Conclusions

The preliminary survey of the medicinal plants used for the treatment and control of animal diarrhoea in Plateau State revealed an array of plants that could be investigated, and if found useful, such plants could be harnessed and used as potential drug candidates for the production of anti-diarrhoeal remedies for livestock. It is therefore, recommended that further studies be carried out on all the above listed plants to validate their efficacy in the treatment of diarrhoea.

## Competing interests

The authors declare that they have no competing interests.

## Authors' contributions

NVO conceived the design of the survey and questionnaire, participated in the survey, and plant sample collection, and drafted the manuscript. SM, participated in the design of the questionnaire, administration of the questionnaire, plant sample collection, was responsible for the compilation of the results and review of the manuscript. ILE, participated in the design of the questionnaire, questionnaire administration, plant sample collection, drafted and reviewed the manuscript. MSM, participated in the design of the questionnaire, questionnaire administration, plant sample collection, drafted the manuscript, was responsible for transporting the plant to Zaria for identification and authentication. JGG, contributed in the design of the questionnaire, questionnaire administration, plant sample collection, drafted the manuscript and was responsible for writing the abstract of the publication. CJD contributed in the design of the questionnaire, questionnaire administration, drafting of the manuscript and was specifically assigned writing the materials and methods of this article. OOO contributed in the design of the questionnaire, administration of the questionnaire, plant sample collection and drafting of the manuscript. ASL, contributed in the designing of the questionnaire, drafting and reviewing of the manuscript. DS contributed in designing of the questionnaire, drafting and reviewing of the manuscript.

All authors thoroughly read and approved of the final manuscript.
